# Molecular Glues: Capable Protein-Binding Small Molecules That Can Change Protein–Protein Interactions and Interactomes for the Potential Treatment of Human Cancer and Neurodegenerative Diseases

**DOI:** 10.3390/ijms23116206

**Published:** 2022-06-01

**Authors:** Fengzhi Li, Ieman A. M. Aljahdali, Xiang Ling

**Affiliations:** 1 Department of Pharmacology & Therapeutics, Roswell Park Comprehensive Cancer Center, Buffalo, NY 14263, USA; ieman.aljahdali@roswellpark.org (I.A.M.A.); xiang.ling@roswellpark.org (X.L.); 2 Developmental Therapeutics (DT) Program, Roswell Park Comprehensive Cancer Center, Buffalo, NY 14263, USA; 3 Gastrointestinal Translational Research Group (GI TRG), Roswell Park Comprehensive Cancer Center, Buffalo, NY 14263, USA; 4 Department of Cellular & Molecular Biology, Roswell Park Comprehensive Cancer Center, Buffalo, NY 14263, USA; 5 Canget BioTekpharma LLC, Buffalo, NY 14203, USA

**Keywords:** molecular glue (MG), small-molecule compounds, human disease, cancer, neurodegenerative disease, protein ubiquitination, protein degradation, E3 ligase protein complex

## Abstract

Molecular glue (MG) compounds are a type of unique small molecule that can change the protein–protein interactions (PPIs) and interactomes by degrading, stabilizing, or activating the target protein after their binging. These small-molecule MGs are gradually being recognized for their potential application in treating human diseases, including cancer. Evidence suggests that small-molecule MG compounds could essentially target any proteins, which play critical roles in human disease etiology, where many of these protein targets were previously considered undruggable. Intriguingly, most MG compounds with high efficacy for cancer treatment can glue on and control multiple key protein targets. On the other hand, a single key protein target can also be glued by multiple MG compounds with distinct chemical structures. The high flexibility of MG–protein interaction profiles provides rich soil for the growth and development of small-molecule MG compounds that can be used as molecular tools to assist in unraveling disease mechanisms, and they can also facilitate drug development for the treatment of human disease, especially human cancer. In this review, we elucidate this concept by using various types of small-molecule MG compounds and their corresponding protein targets that have been documented in the literature.

## 1. Introduction

The term “molecular glue (MG)” has been employed to describe anchoring and scaffolding proteins for spatiotemporally restricting and organizing various types of kinase and/or phosphatase signaling cascades in cells [1]. However, the scope of MG in this review will be within the small (chemical) molecule that can selectively promote the proximity and interaction of two or more proteins [2]. As a result, the MG-induced protein proximity can either be stabilized/activated or destabilized/inactivated, which is dependent on the property of the small molecules and the involved proteins. The MG small molecules such as the plant hormones, auxin [3] and jasmonate [4], and the immunomodulatory imide drugs (IMiDs), thalidomide [5], and lenalidomide (Revlimid) [6,7,8] belong to this category. In contrast, the proteolysis-targeting chimera (PROTAC) small molecules are the artificially designed MG compounds containing two parts: one part (ligand) to glue/bind to a protein neosubstrate and the other part (warhead) to glue/bind to an E3 ligase, where the two parts combine with a chemical link [9]. In terms of the induction of protein degradation, small-molecule MG degrader compounds have some advantages over PROTAC compounds such as lower molecular weight, which makes entry into the cell easier and also works in a substoichiometric manner. For the PROTAC area, researchers can reference recent review articles such as these two citations [10,11]. In this review, we focus on the small (chemical) molecule MGs that either degrade or stabilize protein–protein interactions (PPIs) and interactomes after their binding. We use various model examples to elucidate this concept. To keep the review article relatively succinct, we focus on elucidating different working models without describing the detailed molecular interaction mechanism including the binding ternary interface residue contacts in individual working models. For such ternary interface interaction details, readers could refer to recent review articles [9,12]. In this way, we can let our classified models fit broader research. Finally, citation of appropriate review articles or original publications are based on our discretion for the purpose of providing a succinct overview.

## 2. Molecular Glues for Cancer Treatment

There are many excellent review articles that have systematically reviewed the key findings related to molecular glue (MG) for MG-targeted protein degradation, among others [2,9,12]. Our goal in this review is to emphasize various mechanistic working models while avoiding detailed mechanistic information. Therefore, when a working model includes many original publications, which have been sufficiently reviewed, we collectively draw out the conclusive working models from relevant reviews by citing the review article(s) without citing the original research papers, to keep our review concise.

### 2.1. CRBN-Involved CRL4 Complex-Mediated Degradation of MG-Targeted Proteins

**Figure 1:**
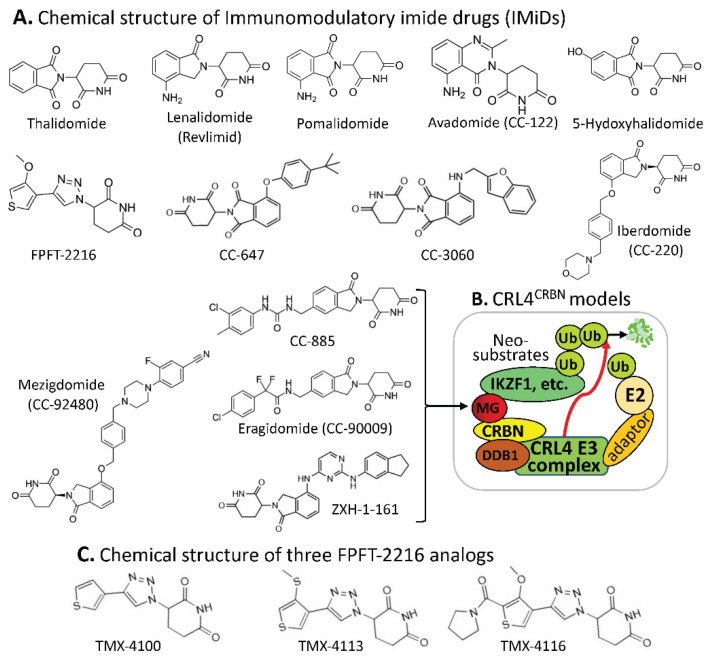
(**A**) The IMiD-type MG compounds’ chemical structures; (**B**) the substrate receptor CRBN-involved E3 ligase protein complex model. Each compound in (**A**) was documented to engage the same E3 ligase protein complex (CRL4^CRBN^) to polyubiquitinate the compound’s neosubstrates shown in Table 1. Then, the polyubiquitinated neosubstrates/protein targets in Table 1 would be degraded through the ubiquitination proteasome pathway; and (**C**) chemical structure of FPJFT-2216-derived three new small molecules (TMX-4100, TMX-4113, TMX-4116).

Human CRBN (cereblon) was originally identified as a candidate protein factor for an autosomal recessive form of mild mental retardation. Relevant to the ubiquitination and degradation of MG-targeted proteins, CRBN is the substrate receptor of the CRL4 (cullin 4-RING E3 ligase) complex, which may also include damaged DNA binding protein 1 (DDB1), among others (Figure 1B). Based on the studies documented in the literature, particular protein members in the CRL4 complex depend on the property of specific MG compounds and may depend on specific, targeted proteins for ubiquitination (examples are shown later). This CRL4^CRBN^ complex through CRBN can be engaged in ubiquitinating many types of proteins for proteasome degradation.

It has been reported that the CRL4^CRBN^ complex E3 ligase can be used by the small-molecule MG of thalidomide and its structural analogs (lenalidomide/Revlimid, pomalidomide, avadomide, 5-hydroxythalidomide, FPFT-2216, CC-647, CC-3060, iberdomide, mezigdomide, CC-885, eragidomide, and ZXH-1-161) to ubiquitinate the MG-recruited/bound protein neosubstrates (Table 1), and these have been well-reviewed recently [12] (Figure 1A,B). However, based on the literature-documented studies, one interesting phenomenon that may be worthy of emphasis is that thalidomide and its individual analogs with different chemical structures can share the same CRL4^CRBN^ complex E3 ligase for ubiquitinating different protein neosubstrates glued by them. Specifically, a single MG compound can glue/bind to multiple different neosubstrate protein targets for CRL4^CRBN^ ubiquitination, while the same protein target/neosubstrate can also be glued by thalidomide and multiple thalidomide analogs (Table 1).

We anticipate that neosubstrate protein targets for these MG compounds will be found in the future. For example, Teng et al. recently demonstrated that FPFT-2216 (Figure 1A) and its chemically modified analogs TMX-4100 and TMX-4113 but not TMX-4116 (Figure 1C) could selectively degrade PDE6D (Phosphodiesterase 6D) [13] through a similar approach as shown in Figure 1B.

The observation summarized in Table 1 has revealed an important concept for further exploration in the research community. That is, as long as the neosubstrate protein targets bound by an MG compound have good specificity, multiple protein targets that can be glued by a single compound may not be an issue with regard to potential toxicity in normal tissues. A good example of this notion is that lenalidomide (Revlimid, Figure 1A) can glue at least 13 neosubstrate protein molecules (Table 1) to be ubiquitinated by CRL4^CRBN^ for proteasome degradation (Figure 1A,B). Revlimid has been successfully commercialized without serious toxicity issues that could block its approval by the FDA. In fact, Revlimid has become one of the most successful anticancer drugs for the treatment of human multiple myeloma (MM) in the clinic. In this regard, it is likely that small-molecule MG compounds that can specifically glue multiple oncogenic proteins for degradation and/or inhibition of their oncogenic activity may have greater potential to be developed into an anticancer drug. Based on previously documented examples, most anticancer drugs that failed in the later phase II and phase III clinical trials were due to their insufficient antitumor efficacy but not due to their toxicity. This is consistent with the fact that most anticancer drug phase I clinical trials were successful, and researchers who performed the studies often concluded that the drug is well-tolerated and warranted further phase II clinical studies.

**Figure 2:**
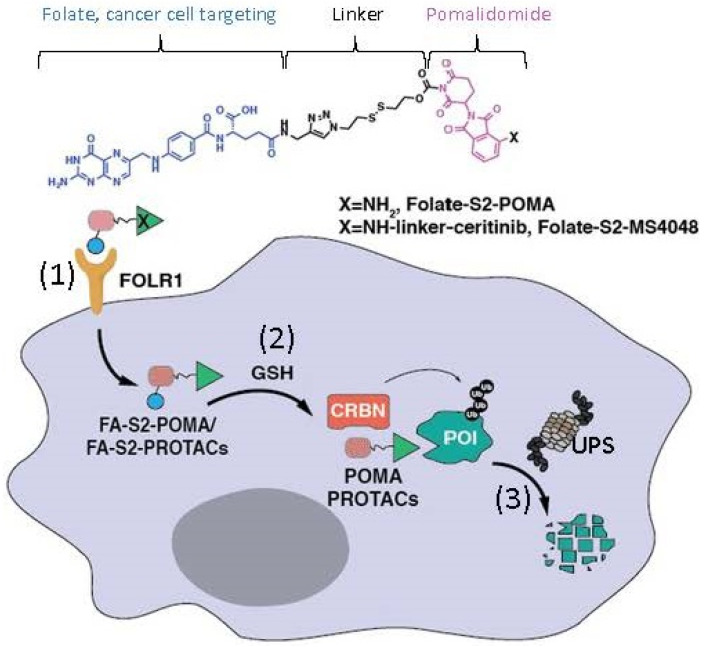
This diagram shows the mode of action of folate–pomalidomide and folate-conjugated pomalidomide-based PROTACs. Upon binding FOLR1 on the cell membrane (1), folate-pomalidomide or folate-conjugated IMiD-based PROTACs are transported into cells, and the active pomalidomide or PROTACs are released after the reduction by endogenous GSH (2). The active pomalidomide or PROTACs recruit endogenous CRBN E3 ligase (similar to the model shown in Figure 1B), leading to polyubiquitination and subsequent degradation of the glued proteins of interest (POIs, neosubstrates) by the UPS (3). This figure is adapted from an original paper by Chen et al. [14].

Additionally, Chen et al. recently reported a modified MG model derived from a pomalidomide-based compound [14]. Specifically, these authors linked a folate group to the MG pomalidomide or the pomalidomide-based PROTAC as prodrugs to specifically recognize the folate receptor α (FOLR1)-positive cancer cells (Figure 2). These authors demonstrated that such prodrugs target FOLR1-positive cancer cells and release the folate-tethered MG/PROTAC, which then degrade the protein targets in FOLR1-positive cancer cells in a CRBN- and proteasome-dependent manner, but this did not occur in FOLR1-negative cancer cells [14]. This is an interesting drug design that extends our views on MG/PROTAC drugs to more specifically target the target-/bait-expressed cancer cells but not target-/bait-negative normal cells by using an additional cancer cell surface target as a bait. However, we should always keep in mind that if an MG/PROTAC drug’s cellular protein target/bait can express in both bait-positive and bait-negative cancer cells, an MG/PROTAC drug designed in this way would not be able to degrade their cellular protein targets in the bait-negative cancer cells. In short, additional considerations are necessary when designing such prodrugs for cancer treatment.

### 2.2. DCAF15-Involved CRL4 Complex-Mediated Degradation of MG-Targeted Proteins

**Figure 3:**
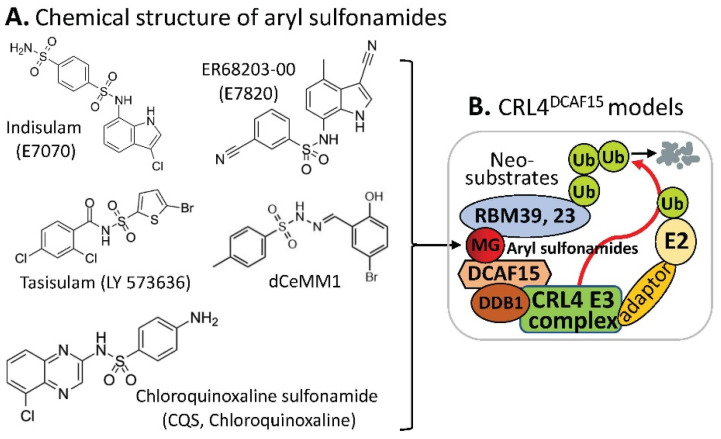
(**A**) The aryl sulfonamide type MG compounds’ chemical structures; (**B**) the substrate receptor DCAF15-involved E3 ligase protein complex model. Each compound in (**A**) was documented to engage the same E3 ligase protein complex (CRL4^DCAF15^) to polyubiquitinate the compound’s neosubstrates shown in Table 2. Then, the polyubiquitinated neosubstrates/protein targets in Table 2 would be degraded through the ubiquitination proteasome pathway.

Human DDB1 and Cullin 4-associated factor 15 (DCAF15) protein is encoded by the *DCAF15* gene, and a recent study revealed that genetic disruption of DCAF15 strongly sensitized cancer cells to natural killer (NK)-mediated clearance [15]. Similar to the case of the CRL4^CRBN^ complex E3 ligase in which CRBN acts as a substrate receptor of CRL4, DCAF15 can also be a part of the CRL4 complex E3 ligase, acting as the CRL4 E3 ligase complex’s substrate receptor/adaptor to be glued on protein neosubstrates, RBM39 and/or RBM23 through the MG compounds, aryl sulfonamides (e.g., indisulam, E7820, tasisulam, dCeMM1, CQS, etc.) for RBM39 and/or RBM23 to be ubiquitinated for proteasome degradation (Figure 3). The details in these studies have been recently reviewed [9,12].

Similar to the observation revealed from the data summarized in Table 1, the CRL4^DCAF15^-based MG aryl sulfonamides can share protein neosubstrates and vice versa; a protein substrate can share MG aryl sulfonamides [12] (Table 2).

Recently, two papers reported that the RNA splicing regulator RBM39 is important for Myc-driven neuroblastoma survival [16,17]. The indisulam-mediated degradation of RBM39 needs the presence of a high-level DCAF15 expression and is highly efficacious against neuroblastoma, leading to significant responses in multiple high-risk disease models, without overt toxicity [16]. Furthermore, in neuroblastoma models, indisulam induces rapid loss of RBM39, accumulation of splicing errors, and the growth inhibition in a DCAF15-dependent manner with metabolome perturbations as well as the mitochondrial dysfunction [17]. Remarkably, complete tumor regression was observed in xenograft models and the Th-MYCN transgenic model of neuroblastoma after intravenous indisulam treatment daily at a dose of 25 mg/kg for 8 days (xenograft mouse model) or for 7 days (Th-MYCN mouse model) [17]. These authors indicated that the dose of 25 mg/kg used in the studies is equivalent to the clinically tolerated dose range of 500–700 mg/m^2^ reported in adult humans [17]. Meanwhile, these authors further confirmed RBM39 loss, RNA splicing, and metabolic changes in vivo after indisulam treatment [17].

Most recently, just before submitting this article for publication, Gosavi et al. reported their interesting findings on the neosubstrates GSPT1 and RBM39 mutation-induced MG drug resistance [18]. Specifically, these authors used the CRISPR-suppressor scanning approach and identified mechanistic classes of CC-885 and ZXH-1-161 resistance mutations in GSPT1, as well as E7820 and Indisulam resistance mutations in RBM39; the mutations in GSPT1 and RBM39 are resistant to the CRBN- (Figure 1) and DCAF15 (Figure 3)-involved E3 ligase-mediated GSPT1 and RBM39 ubiquitination for degradation [18]. These authors found that, while many mutations directly alter the ternary complex heterodimerization surface, distal resistance sites were also identified. CC-885 resistance mutations altered the GSPT1 β-hairpin structural degron and impaired GSPT1 degradation, while E7820 resistance mutations in different domains of RBM39 were operated via distinct mechanisms. The studies further indicated that mutations distal to the RBM39 RRM2 helix 1 structural degron altered maximum levels of RBM39 degradation to abrogate E7820 cytotoxicity, and that resistance mutation sites across targeted protein degradation targets exhibit low levels of sequence conservation [18]. Together, these authors summarized that their study identifies common resistance mechanisms for MG degraders and outlines a general approach to survey neosubstrate requirements necessary for effective degradation [18].

### 2.3. Substrate Receptor-Independent E3 Ligase-Mediated Degradation of Cyclin K by MG

**Figure 4:**
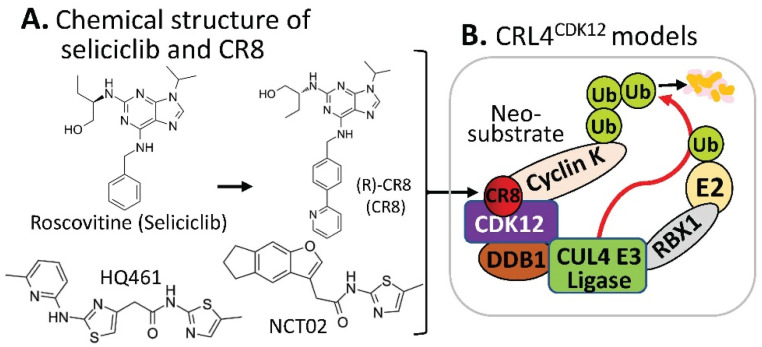
(**A**) The chemical structure of roscovitine/Seliciclib, CR8, HQ461, and NCT02 as MG compounds; (**B**) the substrate receptor-independent E3 ligase protein complex model for ubiquitination of the neosubstrate cyclin K. The MG compound CR8 shown in (**A**) was found through a substrate receptor-independent manner (neither CRBN nor DCAF15 being involved) to glue CDK12–cyclin K directly on DDB1–CUL4 E3 ligase complex to polyubiquitinate cyclin K. Then, the polyubiquitinated cyclin K would be degraded through the ubiquitination proteasome pathway. HQ461 and NCT02 may use a mechanism similar to CR8.

The CDK inhibitor, purine (R) roscovitine (also called CYC202 or Seliciclib) had been used in some clinical trials [19], and recently, a phase II clinical trial with cystic fibrosis patients was completed, which has been reported on the “clinicaltrials.gov” website at https://clinicaltrials.gov/ct2how/NCT02649751. Based on the (R)-roscovitine chemical structure, Ounata et al. synthesized a series of (R)-roscovitine analogs including a compound called (R)-CR8 (Figure 4A) [20]. Further studies indicated that (R)-CR8 selectively inhibits CDK1/2/3/5/7/9 among the 108 kinases tested and is more potent than (R)-roscovitine at inhibiting these kinases and at inducing apoptotic cell death parameters (MTS reduction, 40-fold; lactate dehydrogenase release, 35-fold; caspases activation, 68-fold; and PARP cleavage, 50-fold). Generally speaking, (R)-roscovitine works at a low µM level, while (R)-CR8 works at a low nM level. Convincingly, the improved cell death-inducing activity of (R)-CR8 over (R)-roscovitine was observed in 25 different cancer cell lines including colon (HCT116, LS174T), hepatoma (Huh7, F1) and neuroblastoma (SH-SY5Y) [21].

Intriguingly, by using a molecular barcoding method called PRISM that was previously developed with comprehensive resources (compounds and cell lines) [22,23,24], and through systematically mining databases for correlations between the cytotoxicity of 4518 clinical and preclinical small molecules and the expression levels of E3 ligase components across hundreds of human cancer cell lines, Stabicki et al. identified (R)-CR8 (CR8) as a molecular glue degrader compound to deplete cyclin K [25]. These authors found that CR8-induced degradation of cyclin K is dependent on DDB1 and CDK12 [25]. Specifically, CR8 binds to the CDK12 ATP pocket site to form CDK12-CR8; then the CDK-bound form of CR8 has a solvent-exposed pyridyl moiety that induces the formation of a complex between CDK12-cyclin K and the CUL4 adaptor proteins, DDB1 and RBX1 (RING-box protein 1), bypassing the requirement of a canonical substrate receptor (e.g., not requiring CRBN or DCAF15) and presenting cyclin K for ubiquitination and degradation (Figure 4B). In other words, CR8-engaged CDK12–cyclin K is recruited to the CUL4–RBX1–DDB1 ligase core through DDB1, and CR8 tightens the complex sufficiently to drive CR8-induced ubiquitination and then degradation of cyclin K in the absence of a canonical substrate receptor [25] (Figure 4B). These authors believe that their studies demonstrated that chemical alteration of surface-exposed moieties can confer gain-of-function glue properties to an inhibitor [25].

**Figure 5:**
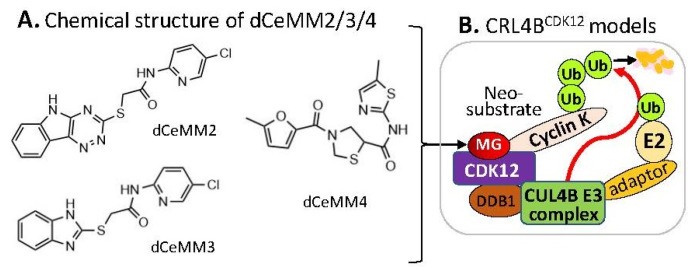
(**A**) The chemical structure of dCeMM2, dCeMM3, and dCeMM4 as MG compounds; (**B**) the substrate receptor-independent E3 ligase protein complex model for ubiquitination of the neosubstrate cyclin K. The MG compounds dCeMM2/3/4 shown in (**A**) was found through a substrate receptor-independent manner (neither CRBN nor DCAF15 being involved) to glue CDK12-cyclin K directly on DDB1–CUL4B E3 ligase complex to polyubiquitinate cyclin K. Then, the polyubiquitinated cyclin K would be degraded through the ubiquitination proteasome pathway.

Alternatively, by using a different approach/strategy based on a 2000 cytostatic/cytotoxic small-molecules (PLACEBO in-house collection) screening in hyponeddylated cells coupled to a multi-omics (proteomics, RNA-Seq, etc.) target deconvolution campaign, Mayor-Ruiz et al. found several chemical structural distinct compounds (dCeMM2, dCeMM3, dCeMM4, Figure 5A) acting as MG degraders that could also induce cyclin K degradation in a low µM level [26]. Their studies indicated that dCeMM2/3/4 compounds-induced cyclin K degradation is mediated via a CRL4B ligase complex without the need for a substrate receptor (e.g., CRBN or DCAF15, Figure 5B), and thus functionally segregating this mechanism from MG degraders that need a canonical substrate receptor (SP) [26]. More details were recently reviewed by Dong et al. [9].

Furthermore, by employing a loss-of-function and gain-of-function genetic screening in human cancer cells, followed by biochemical reconstitution, Lv et.al found a new MG (named HQ461, Figure 4A) that could degrade cyclin K [27] in a mechanism very similar to that of CR8 [25] (Figure 4B). More details were recently reviewed [9].

Interestingly, by screening a small-molecule library against primary colorectal cancer (CRC) heterogeneous spheroids, Dieter et al. found a compound named NCT02 with a chemical structure similar to HQ461 (Figure 4A) that acts as an MG degrader to induce ubiquitination of cyclin K (CCNK) and proteasomal degradation of both CCNK and its partner CDK12 [28].

### 2.4. MG-Mediated Protein Homodimerization-Induced Protein Degradation

**Figure 6:**
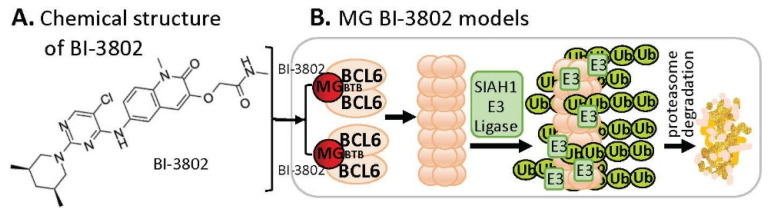
(**A**) The chemical structure of BI-3802 as a special MG compound; (**B**) the diagram model for BI-3802-induced BCL6 polymerization, ubiquitination, and degradation. BI-3802 through the BTB domain of BCL6 makes BCL6 dimerization and then induces the dimerized BCL6 formation of helical filament. The polymerized BCL6 enhances the E3 ligase SIAHI interaction and ubiquitination of the polymerized BCL6 protein for degradation through the ubiquitination proteasome pathway.

B-cell lymphoma 6 (BCL6) is known to be an oncogenic transcriptional factor. High-throughput screening (HTS) with a library of ~1.7 M compounds, followed by carrying out various narrowing-down and alternative confirmation approaches, led to the discovery of a BCL6 MG small-molecule degrader named BI-3802 [29] (Figure 6A). In contrast to the MG compound models that MGs directly glue both their protein neosubstrates and the E3 ligase complex (very similar to PRTOTAC without the linker structure) to induce the glued protein to be polyubiquitinated for proteasome degradation, studies with BI-3802 revealed that the BCL6 monomer protein molecule can be dimerized by the small-molecule MG BI-3802 through the broad-complex, tramtrack, and bric-a-brac (BTB) domain of BCL6 and then assembled into BCL6 filaments, which then facilitates to induce BCL6 ubiquitination by E3 ligase SIAH1 for BCL6 degradation via the proteasome pathway [30] (Figure 6B).

### 2.5. MG-Mediated Protein Heterodimerization-Induced Protein Stabilization for Cell Death

**Figure 7:**
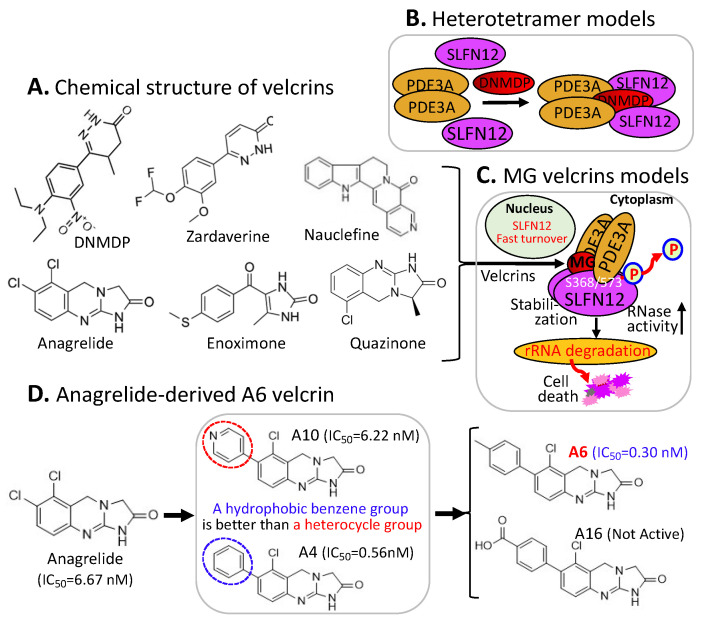
(**A**) Chemical structure of the velcrin-type of MG compounds; (**B**) DNMDP-mediated PDE3A–SLFN12 heterotetramer formation models; (**C**) a cartoon model for elucidating the velcrin compound-induced mechanism of action for inducing cell death; (**D**) summary of the structure–activity relationship (SAR) of representative anagrelide analogs for deriving the lead compound A6. (**D**) is adapted from an original publication by Chen et al. [33].

The small molecule 6-(4-(Diethylamino)-3-nitrophenyl)-5-methyl-4,5-dihydropyridazin-3(2*H*)-one (DNMDP) is known to induce complex formation between phosphodiesterase 3A (PDE3A) and Schlafen family member 12 (SLFN12) and selectively kills cancer cells showing high expression of PDE3A and SLFN12. However, the detailed mechanism is not fully elucidated. Garvie et al. demonstrated that DNMDP, one of a set of structurally distinct small-molecule compounds collectively named by these authors as velcrins (Figure 7A), binds to the constitutive PDE3A dimer and forms an adhesive surface for SLFN12. Then, PDE3A and SLFN12 form a heterotetramer stabilized by the binding of DNMDP [31] (Figure 7B). As a result, the DNMDP-bound tetramer is further stabilized by interactions between SLFN12 and DNMDP [31] (Figure 7B). These authors further demonstrate that SLFN12 is an RNase, that PDE3A binding increases SLFN12 RNase activity, and that SLFN12 RNase activity is required for DNMDP response [31].

It is known that the canonical function of PDE3A is to hydrolyze the phosphodiester bonds in second messenger molecules, such as cAMP and cGMP. In this regard, the finding by Garvie et al. is a phosphodiesterase-activity-independent role for PDE3A [31]. Yan et al. further demonstrated that such noncanonical protein–protein interactions (PPIs) between PDE3A and SLFN12 upon treatment of cancer cells with velcrins (DNMDP, zardaverine, anagrelide, enoximone, quazinone, Figure 7A) resulted in SLFN12 dephosphorylation on Ser368/Ser573 and stabilization, thus increasing SLFN12 RNase activity to degrade rRNA (e.g., 18S and 28S rRNA) and inducing cancer cell deaths [32] (Figure 7C). Consistently, by using a molecular barcoding method called PRISM to screen 4518 drugs against 578 cancer cell lines in pools, followed by confirmation, Corsello et al. found that a number of compounds including anagrelide (PDE3A inhibitor), DCEBIO (K^+^ channel activator), AG-1296 (kinase inhibitor) and tanaproget (PGR agonist) kill cancer cells by stimulating PDE3A–SLFN12 interaction [23]. Together, the finding discussed above [31,32] revealed a unique mechanism by which velcrins induce cancer cell death through the MG-compound-mediated stabilization of PDE3A–SLFN12 complex formation in a gain-of-function manner.

Additionally, Chen et al. recently reported a high-resolution cryo-electron microscopy structure of PDE3A–SLFN12 complexes isolated from cultured HeLa cells pre-treated with either anagrelide, nauclefine, or DNMDP [33]. Importantly, based on the complex structure and through computer algorism design of anagrelide analogs, followed by synthesis and testing of some of these anagrelide-derived analogs, an A6 compound (Figure 7D) with an enhanced SLFN12 interaction achieved superior antitumor efficacy in tumor xenografts in an intragastric route with a schedule of administration of A6 (5 mg/kg) once per day for 8 days, then twice per day for 6 days without showing toxicity (no body weight loss shown) [33].

### 2.6. The Ligand of a PROTAC-Type MG Binding to More than One Protein for Degradation

**Figure 8:**
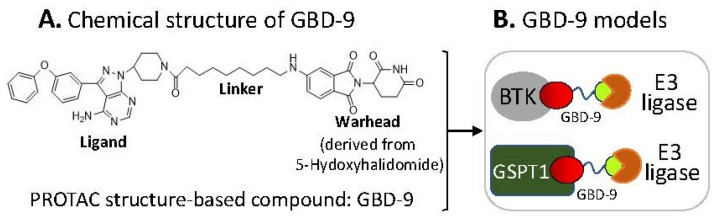
(**A**) Chemical structure of the GBD-9 compound; (**B**) cartoon models for the GBD-9 ligand part to find two distinct neosubstrate proteins (BTK, GSPT1) for polyubiquitination by E3 ligases. Then, the polyubiquitinated proteins would be degraded through the ubiquitination proteasome pathway.

There is a recent report which shows that a PROTAC structure-based small-molecule GBD-9 (Figure 8A) can act as a PROTAC to degrade Bruton’s tyrosine kinase (BTK) and can also act as a molecular glue to degrade G1-to-S phase transition 1 (GSPT1) proteins [34]. Given that BTK and GSPT1 function in distinct biological processes, this suggests that the ligand part of GBD-9 basically glues/binds to both BTK and GSPT1 proteins independently (Figure 8B). Given that the previously documented one MG can bind to multiple target proteins and one target protein can be glued by multiple MGs (Table 1), it is not surprising that the GBD-9 ligand recruits two different proteins (BTK, GSPT1) for subsequent ubiquitination and degradation. Furthermore, based on the current studies, we predict that it is likely that most (if not all) MG and PROTAC small-molecule compounds could selectively bind to/glue on more than one neosubstrate target protein, which would be a productive area to pursue further studies using the existing MG and PROTAC small-molecule compounds to expand our current knowledge.

### 2.7. MG Stabilization of Weak Protein–Ubiquitin Interactions for Function Inhibition or Activation

**Figure 9:**
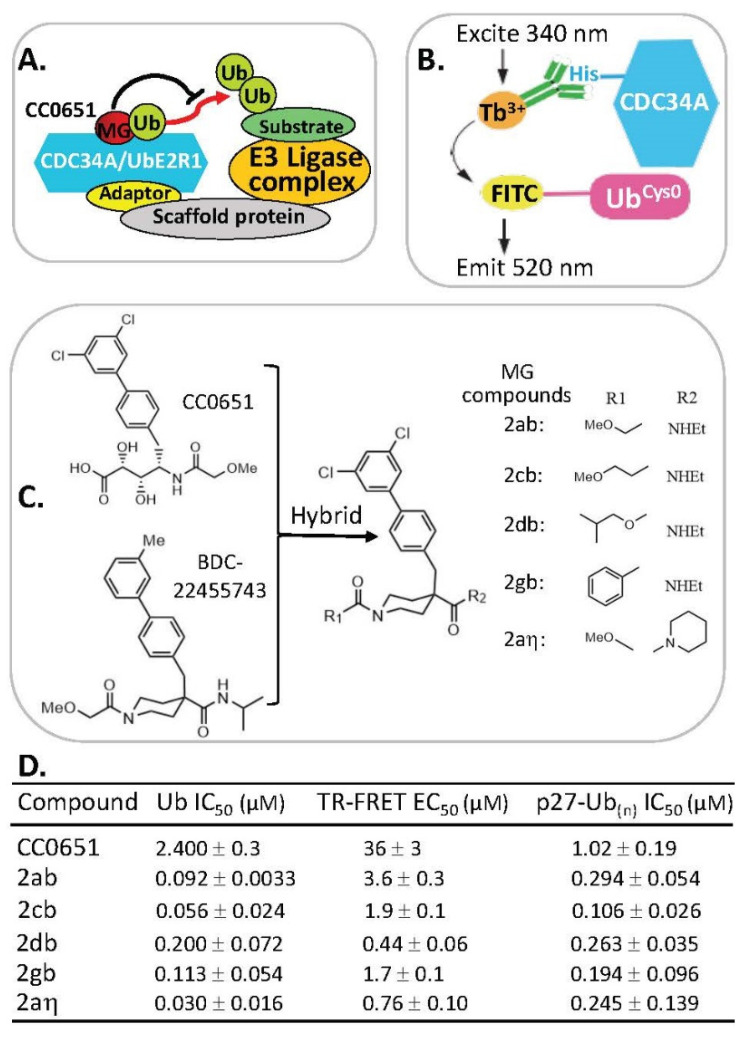
(**A**) Cartoon models of CDC34A and E3 ligase-mediated substrate ubiquitination being blocked by MG CC0651 (i.e., inhibition of ubiquitin transfer by stabilizing the noncovalent CDC34A-donor ubiquitin complex); (**B**) schematic of TR-FRET assay to detect CDC34A/UbE2R1–ubiquitin interactions. The assay used an N-terminal His-tagged CDC34A for recognition by an anti-His6 antibody coupled to Tb3+ and an N-terminal cysteine mutant of ubiquitin (denoted UbCys0) stoichiometrically labeled with 5′-iodoacetamide-fluorescein. In response to titration with CC0651, excitation of Tb3+ at 340 nm resulted in fluorescence energy transfer to the fluorescein moiety and emission at 520 nm; (**C**) diagram to explain the led compounds 2ab, 2cb, 2db, 2gb, and 2aη generated by medicinal chemistry hybridization of the prototype compound CC0651 and the isonipecotamide hit BDC22455743; (**D**) Ub IC_50_ (µM), TR-FRET EC_50_ (µM), and p27-Ub_(n)_ IC_50_ (µM) for the prototype compound CC0651 and the led compounds 2ab, 2cb, 2db, 2gb, and 2aη were shown. IC_50_ values represent the mean +/− variance, *n* = 2. EC_50_ values represent the mean +/− SD, *n* = 3. (**B**,**D**) are adapted from an original paper by St-Cyr et al. (some information is also from their provided supplemental materials) [37].

Previous studies indicated that the CC0651 compound stabilizes a weak interaction of the donor ubiquitin-binding site of CDC34A (also called UbE2R1) with ubiquitin [35] (Figure 9A). However, the CC0651 in vitro potency is modest, and its efficacy in cells is poor [36]. Based on this CC0651 prototype molecular glue (MG), a time-resolved Forster resonance energy transfer (TR-FRET)-based assay (Figure 9B) was developed and used to screen a total of 995 structurally diverse chemical compounds. These compounds were chosen from an Asinex protein–protein interaction (PPI) library of 10,496 compounds through 2D fingerprinting clustering (221), computer docking (626), and structure uniqueness-based selection (148) in parallel with CC065 as positive controls and DMSO as negative controls [37].

One hit compound (BDC22455743) shared chemical structure similarity to CC0651 that could stabilize the noncovalent complex between CDC34A and ubiquitin to inhibit ubiquitin transfer to the substrate (Figure 9A,C). Through a series of medicinal chemistry synthetic strategy to generate BDC22455743-based analogs in parallel with TR-FRET assay testing, these authors found that a CC0651 and BDC22455743 hybrid core compound (Figure 9C)-based derivatives exhibits a stronger ubiquitin (Ub) IC_50_ (0.03–0.2 µM) and TR-FRET EC_50_ (0.44–3.6 µM) for compounds 2ab, 2cb, 2db, 2gb, and 2aη versus Ub IC_50_ 2.4 µM for CC0651 and TR-FRET EC_50_ 36 µM for CC0651 [37] (Figure 9C,D). These findings lay a foundation for further studies of the antitumor activity and efficacy in vitro and in vivo for the lead compounds. We look forward to new publications in this area from these authors in the coming years.

There are other MG small molecules that can enhance either neosubstrate protein-E3 ligase interactions (e.g., β-catenin with its cognate E3 ligase and SKP1^β-TrCP^ [38]) or protein–protein interactions (e.g., 14-3-3 with its partner ChREBP [39]), which have been well-reviewed under the section of “Gluing weak interactions” in a recent review article [12]. For the sake of brevity, we will not discuss that review any further.

### 2.8. MG Covalently Engaged/Bound to E3 Ligase and Recruited TP53/p53 for p53 Activation

**Figure 10:**
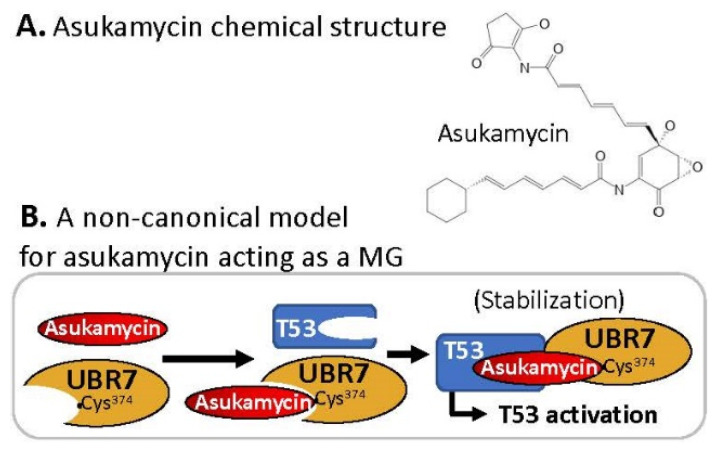
(**A**) Chemical structure of the non-canonical MG compound asukamycin; (**B**) asukamycin-mediated covalently engaged TP53/p53-UBR7 formation models.

This is a special case, and we are not sure this case can be classified into the canonical MG. Specifically, the MG asukamycin (Figure 10A) at a 1–50 µM range engaged/bound to the putative E3 ligase UBR7 by using its electrophilic site to covalently link to the UBR7-Cys374 site and at the same time glued and stabilized/activated the neosubstrate TP53 tumor suppressor transcription activity in a UBR7-dependent manner [40] (Figure 10B). As a result, asukamycin exhibited the inhibition of cancer cell growth across 250 cancer cell lines with an IC_50_ in a range of 5–30 µM [40]. However, given that asukamycin has multiple electrophilic sites, it would be intriguing to determine whether asukamycin could covalently glue other E3 ligases and/or other neosubstrates, not only TP53.

### 2.9. FL118, as an MG, Binds to Dephosphorylates and Degrades Oncogenic Protein DDX5

**Figure 11:**
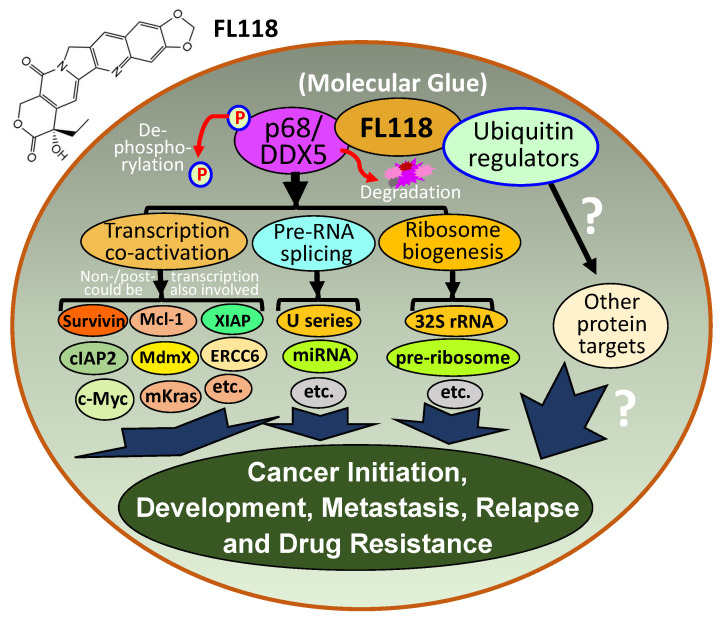
FL118 mechanism of action (MOA). DDX5 is a multifunctional master regulator involved in (1) co-activation of transcription of many oncogenes through the direct interactions of different transcription factors (e.g., c-Myc) in the oncogenic gene promoters, (2) regulation of miRNA and pre-RNA splicing (e.g., U1, U2, U3, …, snRNP), and (3) ribosome biogenesis (e.g., 32S rRNA, pre-ribosome). The small-molecule drug FL118 directly binds to and functionally dephosphorylates and degrades DDX5 protein (without decreasing DDX5 mRNA) through the proteasome degradation pathway. This suggests that FL118 could glue both DDX5 and ubiquitin-involved protein stability/degradation regulators (i.e., FL118 acts as a “molecular glue degrader”). All the DDX5 downstream protein targets were known to be involved in cancer initiation, development, metastasis, recurrence, and treatment resistance. Therefore, indirectly blocking DDX5 downstream targets through direct dephosphorylation and degradation of DDX5 by FL118 could result in FL118 high antitumor efficacy as demonstrated in our recent study, which used human CRC and PDAC cell and tumor models [49].

DDX5 (also called p68) is a multifunctional oncogenic DEAD-box RNA helicase and a transcription cofactor. DDX5 is gradually being recognized as a potential therapeutic target and biomarker for cancer treatment [41]. Importantly, the new role of DDX5 in cancer DNA repair could well unify some inconsistencies reported in the literature and makes DDX5 a superior cancer therapeutic target and biomarker [41]. On the other hand, a small molecule FL118 was discovered via HTS [42]. FL118 was found to exhibit high antitumor efficacy by targeting multiple antiapoptotic proteins, including survivin, Mcl-1, XIAP, cIAP2, and MdmX [42,43,44,45,46,47,48]. However, the direct target of FL118 is unknown. In this regard, using the small-molecule drug FL118 affinity column purification of cancer cell lysate proteins, followed by mass spectrometry analyses, Ling et al. identified that the DDX5 protein is the direct biochemical target of FL118. These authors demonstrated that FL118 acting as an MG degrader strongly binds to, dephosphorylates, and degrades DDX5 oncoprotein via proteasome degradation pathway, without decreasing DDX5 mRNA [49]. These studies further revealed that DDX5 is a master regulator for controlling the expression of multiple oncogenic proteins including survivin, Mcl-1, XIAP, cIAP2, c-Myc, and mutant Kras (mKras) [49]. Pancreatic ductal adenocarcinoma (PDAC) cells with DDX5 knockout by vector-free Crispr technology are resistant to FL118 treatment [49], indicating DDX5 is a bona fide FL118 target. Importantly, human tumor animal model studies further indicated that FL118 exhibits high efficacy to eliminate human PDAC and colorectal cancer (CRC) tumors that have a high expression of DDX5, while FL118 exhibits less effectiveness for PDAC and CRC tumors with low DDX5 expression [49]. However, it is currently unknown which protein(s) and protein complex, as well as which ubiquitin regulators are involved in the dephosphorylation, ubiquitination, and degradation of DDX5 by FL118. Nevertheless, based on the literature-documented function of DDX5 plus the new discoveries related to FL118 and DDX5 in our paper [49], we outlined a graphical abstract to explain why FL118 has exceptional antitumor activity (Figure 11).

## 3. Molecular Glues (MGs) for Treating Neurodegenerative Diseases

### 3.1. MG for Treating Huntington’s Disease

**Figure 12:**
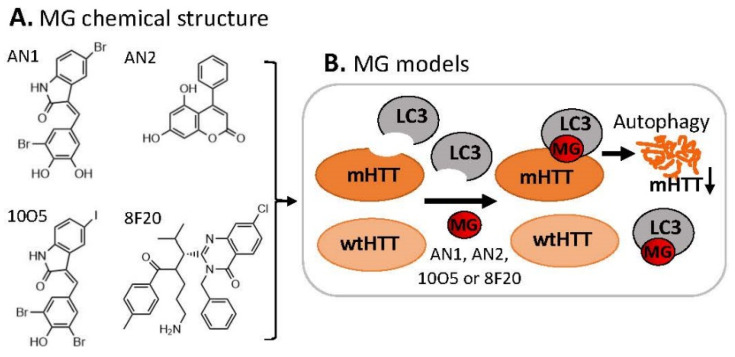
(**A**) Chemical structure of AN1, AN2, 10O5, and 8F20 as MG compounds; (**B**) a cartoon model to show the key finding for disease treatment. Such types of MG discovered by the logically designed assay could only ligate LC3 to mHTT but not wtHTT. Thus, mHTT could be glued on LC3 for degradation of mHTT but not wtHTT by autophagy.

Neurodegenerative disorders are known to be the results of autophagy malfunction because clearance of disease-related mutant proteins is highly dependent on autophagy, for example, extended polyglutamine (polyQ)-containing proteins that cause various neurodegenerative diseases such as Huntington’s disease (HD) [50]. In this regard, Li et al. hypothesized that compounds that interact with both the autophagosome protein microtubule-associated protein 1A/1B light-chain 3 (LC3) and the disease-causing mutant huntingtin protein (mHTT) may target mHTT for autophagic clearance [51]. These authors performed an HTS study of a homemade small-molecule microarray (SMM) printed on phenyl-isocyanate functionalized glass slides containing 3375 bioactive compounds (1527 FDA-approved drugs, 1053 natural products, and 795 known inhibitors) [51]. They identified four compounds (AN1, AN2, 10O5, 8F20, Figure 12A) that interact with both LC3 and mHTT but not with the wild-type HTT (wtHTT) [51] (Figure 12B). These MG compounds (the authors called linkers) can lower mHTT but not wtHTT levels in cultured mouse neurons via autophagy, as well as lower mHTT in cells from HD patients [51]. Using different cell systems, these authors demonstrated that such MG compounds enhance mHTT–LC3 interactions and tether mHTT to autophagosomes. Most importantly, they further showed that these MG compounds rescue HD-relevant phenotypes both in cells and in vivo (HD flies and mouse brains) [51].

### 3.2. Other Neurodegenerative Diseases

**Figure 13:**
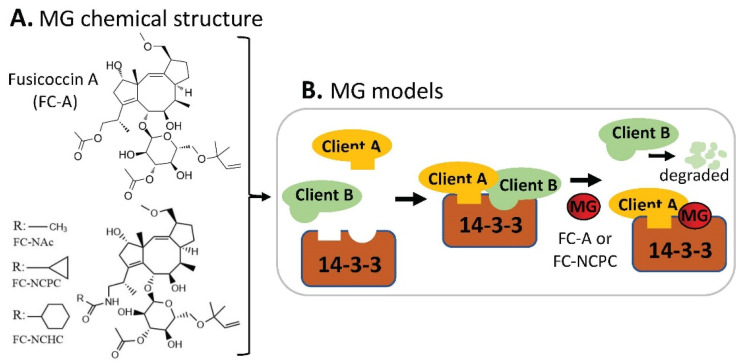
(**A**) Chemical structure of fusicoccin A (FC-A), FC-NAc, FC-NCPC, and FC-NCHC as MG compounds; (**B**) a cartoon model to show the key finding for potential disease treatment. This type of MG compound could strengthen some 14-3-3 clients’ binding to 14-3-3, while disrupting other clients’ binding on 14-3-3 as shown in (**B**).

By using E18 rat cortical neurons grown in 96-well plates and through high-content imaging to screen 30 analogs derived from the prototype 14-3-3 protein–protein interaction (PPI) modulator fusicoccin-A (FC-A), Kaplan et al. found that FC-NCHC, FC-NCPC, and FC-NAc are the top 3 MG compounds (Figure 13A) with the most potential to stimulate neurite outgrowth [52]. Further studies indicated that FC-NCPC enhances axon regeneration in vitro, and both FC-A and FC-NCPC affect the 14-3-3 interactome by stabilizing some 14-3-3-client PPIs while disrupting others [52] (Figure 13B). Given that 14-3-3s are abundantly expressed in the central nervous system (CNS), this study suggests that MG compounds that target 14-3-3 PPIs may be developed into drugs for treating neurodegenerative diseases.

## 4. Perspectives and Summary

New MG compounds and new protein targets for the MG compounds documented in the literature will be discovered over time. Given that MG compounds can glue on any key disease-genic proteins with or without classic binding pockets or enzymatic sites, it is expected that there will be no undruggable proteins with the use of MG compounds. Specifically, the binding of proteins by an MG small molecule does not need a protein with binding pockets on the protein superstructure surface. Instead, an MG small molecule can glue on a flexible loose structure presented or even created on the protein surface after the binding of an MG small molecule on the protein target; in turn, the superstructure configuration of the protein will be adapted, and the PPIs and interactomes will be further changed via modification of the protein surface. This will further strengthen the MG function to induce the targeted protein degradation, stabilization, or activation through various molecular mechanisms (Figure 1, Figure 2, Figure 3, Figure 4, Figure 5, Figure 6, Figure 7, Figure 8, Figure 9, Figure 10, Figure 11, Figure 12 and Figure 13), or it can even restore a mutant protein to act like a wild-type protein for serving its correct function [2,12]. As summarized in Table 1 and Table 2, one MG small molecule could bind/glue on different protein targets, while the same protein target could be bound/glued by multiple MG compounds with different chemical structures; this high flexibility would generate various molecular gluing tools for disease mechanism elucidation but would also provide a soil foundation for developing better drugs. In short, from an optimistic point of view and based on the MG property, MG compounds would have the potential for the treatment of various types of human diseases, especially human cancer, through changing PPIs and interactomes after gluing on the target. The key is to have a good idea-based study design with a workable assay/system to find them and then develop them. In other words, the invention of novel drug discovery systems/strategies would likely be a restriction step for finding novel/versatile MGs. One way to speed up this process is to combine computer-aided artificial intelligence (AI) technologies with modern medicinal chemistry technologies plus additional wet lab research efforts. This may, in turn, change the situation of finding MG compounds merely through serendipity. Additionally, although we did not touch on the detailed mechanistic PPI and interactomes in this article, we believe that a better understanding of MG-mediated PPIs and interactomes would be impotent for the discovery and development of novel MG compounds.

Finally, we would like to point out that small molecules such as thalidomide (Figure 1) or FL118 (Figure 11) with a “naked” core chemical structure would have a high potential to create a series of core structure derived analogs by adding one or more chemical group side chains on them. Thus, the derived analogs may be able to glue a different protein in the same or different protein families (Table 1) or glue the same protein molecule with different interactomes [2,12].

## Figures and Tables

**Table 1:** Relationship of MGs with their recruited protein substrates using CRL4^CRBN^ *.

MG Compounds	Neosubstrates/ Protein Targets		Neosubstrates/ Protein Targets	Molecular Glue (MG) Compounds
Thalidomide	IKZF1 ^#^, IKZF3, ZNF692, ZNF276, SALL4, RNF166, ZBTB16, FAM83F, p63		IKZF1	thalidomide, lenalidomide, pomalidomide, avadomide/CC-122, FPFT-2216, iberdomide/CC-220, CC-3060, CC-92480, CC-885
Lenalidomide (Revlimid)	IKZF1, IKZF3, ZFP91, ZFP692, ZNF276, ZNF653, ZNF827, SALL4, RNF166, WIZ1, CK1α, FAM83F, RAB28		IKZF3	thalidomide, lenalidomide, pomalidomide, avadomide/CC-122, iberdomide/CC-220, 92480, CC-885
Pomalidomide	IKZF1, IKZF3, ZFP91, ZFP692, ZNF276, ZNF653, ZNF827, SALL4, RNF166, GZF1, ZBTB39, ZNF98, WIZ1, ZBTB16, FAM83F, RAB28, DTWD1		ZNF692	thalidomide, lenalidomide, pomalidomide,
Avadomide (CC-122)	IKZF1, IKZF3, ZFP91		ZNF276	thalidomide, lenalidomide, pomalidomide,
5-hydroxy-thalidomide	SALL4, ZBTB16		SALL4	thalidomide, lenalidomide, pomalidomide, FPFT-2216,
FPFT-2216	IKZF1, CK1α		RNF166	thalidomide, lenalidomide, pomalidomide,
Iberdomide (CC-220)	IKZF1, IKZF3, ZFP91, ZNF98		ZBTB16	thalidomide, pomalidomide, 5-hydroxythalidomide, CC-647, CC-3060
CC-647	ZBTB16		FAM83F	thalidomide, lenalidomide, pomalidomide,
CC-3060	ZBTB16, IKZF1, ZFP91, ZNF276		p63	thalidomide
CC-92480	IKZF1, IKZF3		ZFP91	lenalidomide, pomalidomide, avadomide/CC-122, iberdomide/CC-220, CC-3060
CC-885	IKZF1, IKZF3, GSPT1, CK1α, PLK1, HBS1L		ZNF653	lenalidomide, pomalidomide,
CC-90009	GSPT1		ZNF827	lenalidomide, pomalidomide,
ZXH-1-161	GSPT1, GSPT2		WIZ1	thalidomide, lenalidomide,
			CK1α	lenalidomide, FPFT-2216, CC-885
			RAB28	lenalidomide, pomalidomide,
			GZF1	pomalidomide,
			ZBTB39	pomalidomide,
			ZNF98	pomalidomide, iberdomide/CC-220,
			DTWD1	pomalidomide,
			ZNF276	CC-3060
			GSPT1	CC-885, CC-90009, ZXH-1-161
			PLK1	CC-885
			HBS1L	CC-885
			GSPT2	ZXH-1-161

* Table 1 data summary is based on the review article written recently by Kozicka and Thoma [12]. ^#^ Abbreviations: **CK1α**, casein kinase 1α; **DTWD1**, DTW domain-containing protein 1; **FAM83F**, family with sequence similarity 83, member F; **GSPT1**, G1-to-S phase transition 1; **GZF1**, GDNF-inducible zinc-finger protein 1; **HBS1L**, HBS1-like translational GTPase; **IKZF1**, IKAROS family zinc-finger protein 1; **PLK1**, polo-like kinase 1; **RAB28**, Ras-associated protein Rab28; **RNF166**, ring-finger protein 166; **SALL4**, Sal-like protein 4; **WIZ1**, widely interspaced zinc-finger protein 1; **ZBTB16**, zinc-finger and BTB domain-containing protein 16; **ZFP91**, zinc-finger protein 91; **ZNF692**, zinc-finger protein 692.

**Table 2:** Relationship of MGs with their recruited protein substrates using CRL4^DCAF15^ *.

Compounds	Protein Targets/Neosubstrates		Protein Targets	MG Compounds
Indisulam	^#^ RBM39, RBM23		RBM39	Indisulam, E7820, Tasisulam, CQS, dCeMM1
E7820	RBM39, RBM23		RBM23	Indisulam, E7820, Tasisulam, CQS,
Tasisulam	RBM39, RBM23			
CQS	RBM39, RBM23			
dCeMM1	RBM39			

* Table 2 data summary is based on the review article written recently by Kozicka and Thoma [12]. ^#^ Abbreviations: **RBM39**, RNA-binding motif protein 39.

## Data Availability

Not applicable.
